# Integrative genomic and spatial transcriptomic analysis elucidates the oligodendrocyte‐mediated etiology of epileptic cortical thinning

**DOI:** 10.1002/epi4.70251

**Published:** 2026-03-26

**Authors:** Dingyuan Zhang, Qianqian Zhang, Guangming Li, Lingting Yu, Yanling Ma, Xiaoli Hong, Yujie Kui, Shanshan Cai, Jianguang Sun, Zechao Zhu

**Affiliations:** ^1^ Department of Neurosurgery Haiyan People's Hospital, Affiliated Haiyan Hospital of Jiaxing University Jiaxing China; ^2^ Division of Biomedical and Life Sciences Faculty of Health and Medicine, Lancaster University Lancaster UK

**Keywords:** cell‐type‐specific Mendelian randomization, cortical thickness, focal epilepsy, genomic structural equation modeling, oligodendrocytes

## Abstract

**Objective:**

Focal epilepsy is characterized by progressive cortical thinning, particularly within limbic structures; however, whether this atrophy reflects acquired seizure‐induced damage or shared genetic predisposition remains unresolved.

**Methods:**

We integrated genome‐wide association study (GWAS) summary statistics from the ILAE Consortium (focal epilepsy: 15212 cases; 29 677 controls), ENIGMA (cortical thickness: *N* = 33 992), and COGENT (cognitive function: *N* = 257 841) using linkage disequilibrium score regression and genomic structural equation modeling (Genomic SEM). A latent cortical factor (F‐EpiCortex) was derived and interrogated through MAGMA gene‐based analysis, cell‐type‐specific Mendelian randomization (csMR) using brain single‐cell expression quantitative trait loci, and spatial transcriptomic mapping (gsMap) across mouse embryonic and human cortical datasets.

**Results:**

Focal epilepsy exhibited significant negative genetic correlations with cingulate cortical thickness (rg = −0.23 to −0.27; *p* < 0.05). Genomic SEM identified a well‐fitting two‐factor model (CFI = 0.916) wherein focal epilepsy genetic liability was associated with reduced cortical thickness (*β* = −0.30; *p* = 0.02), while cognitive function showed a protective association (*β* = 0.10; *p* = 0.04). GWAS of the F‐EpiCortex latent factor identified nine genome‐wide significant loci, with *DPYSL5* (*p* = 1.88 × 10^−11^) as the lead signal. Cell‐type‐specific analysis revealed oligodendrocytes as the predominant cellular mediator, with *DPYSL5* (*β* = −0.21; *p* = 1.3 × 10^−10^) and *SLC16A8* (*β* = −0.28; *p* = 8.9 × 10^−8^) exhibiting robust protective effects predominantly within the oligodendrocyte lineage. Spatial transcriptomic validation confirmed oligodendrocyte enrichment across human cingulate and temporal cortices, with 70% concordance between csMR predictions and spatial expression patterns. Experimental validation in human oligodendrocytes under glutamate‐induced excitotoxic stress demonstrated significant downregulation of the prioritized protective proteins, providing functional evidence for their susceptibility to epilepsy‐associated injury.

**Significance:**

These findings implicate oligodendrocyte dysfunction as a shared genetic component linking focal epilepsy to cortical atrophy. This extends the “scars of seizures” paradigm by supporting a complementary neurodevelopmental origin model, with implications for neuroprotective therapeutic strategies.

**Plain Language Summary:**

Focal epilepsy is often accompanied by a progressive thinning of the brain's cortex, which has traditionally been viewed purely as cumulative damage from repeated seizures. In this study, we investigated whether an underlying genetic predisposition also plays a role. By analyzing large‐scale genetic and brain imaging datasets, we discovered a shared genetic link between focal epilepsy and cortical thinning. Furthermore, we traced this genetic vulnerability specifically to oligodendrocytes—the cells responsible for supporting and insulating nerve fibers. Our findings suggest that cortical thinning is not merely a “scar” from seizures, but partly a preexisting structural vulnerability driven by reduced protective functions of specific genes (such as *DPYSL5* and *SLC16A8*) in these support cells. This offers a new perspective on preventing brain structural changes in epilepsy.

**Key points**

- Genomic SEM reveals focal epilepsy genetic liability drives limbic cortical thinning independent of acquired seizure damage.
- Cell‐type‐specific Mendelian randomization implicates oligodendrocyte dysfunction as the primary cellular mediator.
- DPYSL5 and SLC16A8 emerged as key protective genes within the oligodendrocyte lineage against cortical atrophy.
- Spatial transcriptomics and in vitro excitotoxicity models validate the selective vulnerability of these oligodendrocyte factors.

## 1 INTRODUCTION

Focal epilepsy is characterized by paroxysmal discharges and progressive alterations in cortical morphology and cognition.^1^ While seizure control remains the primary clinical objective, the burden of the disease extends significantly to progressive cortical thinning, particularly within the limbic and paralimbic circuits—most notably the anterior cingulate and temporal cortices.^2^ These structural anomalies are strongly correlated with cognitive comorbidities and psychiatric outcomes, suggesting that the underlying pathophysiology compromises neural architecture beyond the immediate epileptogenic zone.^3^

While recurrent seizures contribute to acquired neurotoxicity, the extent to which structural deficits reflect shared genetic architecture remains unclear. We investigate whether genetic variants predispose to both epileptogenesis and structural vulnerability. Large‐scale neuroimaging consortia, such as ENIGMA‐Epilepsy, have robustly quantified widespread cortical atrophy in focal epilepsy, demonstrating effects that often precede the onset of chronic intractable seizures.^4^ Concurrently, recent genome‐wide association studies (GWAS) have identified risk loci implicated in synaptic plasticity and structural maintenance, hinting at a genetic basis for structural variations.^4^, ^5^ However, standard univariate approaches fail to disentangle whether these morphological changes are secondary sequelae—“scars of seizures”—or manifestations of a pleiotropic “neurodevelopmental origin”.^6^

Resolving this causality dilemma requires advanced multivariate statistical frameworks capable of isolating shared genetic variance components. Genomic structural equation modeling (genomic SEM) offers a powerful methodology to model latent genetic factors that capture shared structural integrity across functionally connected brain regions, independent of direct disease liability.^7^ By defining a latent factor representing intrinsic cortical vulnerability, it becomes possible to interrogate the biological basis of structural compromise without the confounding influence of seizure burden, disease duration, or medication effects.^8^ This approach allows for the separation of genetic pleiotropy from causal mediation, providing a clearer window into the cellular mechanisms governing cortical stability.

In this study, we employed genomic SEM to model a novel latent factor, “F‐EpiCortex,” which captures the shared genetic architecture of the rostral and caudal anterior cingulate (rACC, cACC), posterior cingulate (PCC), and temporal cortex—regions central to the limbic network implicated in focal epilepsy.^9^ We integrated this structural modeling with cell‐specific causal inference mathematical modeling and spatial transcriptomics (gsMap)^10^ to map genetic risks to specific cellular populations. This multidimensional framework allows us to dissect whether cortical atrophy arises from broad disease liability or distinct, cell‐type‐specific developmental failures—such as compromised glial–neuronal interactions. By mapping these genetic risks to specific cellular populations, we aim to reframe cortical thinning not merely as a consequence of seizure toxicity but as a fundamental neurodevelopmental feature of the disorder.

## 2 METHODS

### 2.1 Study design and data sources

We integrated GWAS summary statistics using LDSC, Genomic SEM, MAGMA, cell‐type‐specific Mendelian randomization (csMR), and spatial transcriptomic mapping (gsMap).

GWAS summary statistics were obtained from three primary consortia. For epilepsy, we utilized meta‐analysis data from the International League Against Epilepsy (ILAE) Consortium, comprising 15 212 cases and 29 677 controls for focal epilepsy, and 3769 cases and 29 677 controls for genetic generalized epilepsy (GGE).^5^ For cortical structure, we obtained regional cortical thickness summary statistics from the ENIGMA consortium, which included MRI‐derived measurements for 34 regions (Desikan‐Killiany atlas) across 33 992 individuals of European ancestry.^11^ For cognitive function, we utilized summary statistics from the COGENT consortium meta‐analysis of cognitive performance (*N* = 257 841).^12^

### 2.2 Genetic correlation analysis

To estimate the genetic overlap between epilepsy subtypes and cortical structure, we performed bivariate linkage disequilibrium (LD) score regression (v1.0.1). We utilized pre‐computed LD scores from the 1000 Genomes European reference panel (Phase 3). Analysis was restricted to HapMap3 variants with a minor allele frequency (MAF) >0.01, excluding the major histocompatibility complex region. For epilepsy phenotypes, we applied a liability scale transformation assuming a population prevalence of 0.6% and sample prevalences matching the respective GWAS cohorts. Multiple testing correction was performed using the Benjamini–Hochberg false discovery rate (FDR) method.

### 2.3 Genomic structural equation modeling

To model the latent genetic architecture shared among focal epilepsy, cognitive function, and cortical thickness, we employed genomic structural equation modeling (Genomic SEM; version 0.0.5). To construct a latent factor with maximal statistical power, we employed a data‐driven selection strategy based on the bivariate LDSC results. We specifically included the rostral anterior cingulate (rACC), caudal anterior cingulate (cACC), PCC, inferior temporal cortex (IT), and insula, as these regions demonstrated the strongest genetic overlap with focal epilepsy. This filtering step was designed to enhance the signal‐to‐noise ratio, thereby isolating the shared genetic variance most relevant to the disease liability. While the insula (*λ* = 0.68) and IT cortex (*λ* = 0.63) exhibited comparatively lower factor loadings than the cingulate regions, their inclusion was justified by their well‐established functional connectivity within the limbic network and their documented involvement in focal epilepsy. Sensitivity analyses confirmed that a cingulate‐only model did not significantly alter the downstream userGWAS results or the genetic correlation with focal epilepsy, supporting the robustness of the five‐region specification.

We first estimated the genetic covariance matrix (S) and its sampling covariance matrix (V) using multivariate LDSC. Focal epilepsy was modeled on the liability scale (population prevalence 0.6%), while cognition and cortical measures were analyzed on the observed scale. We subsequently specified and compared three structural models: a single common factor model (Model 1), a two‐factor regression model (Model 2), and an independent paths model (Model 3). Model 1 posited a single latent liability factor across all traits. Model 2, our primary hypothesized model, specified a latent cortical factor (“F‐EpiCortex”) capturing shared variance among the five brain regions, with focal epilepsy and cognition modeled as simultaneous predictors. Models were estimated using diagonally weighted least squares (DWLS) with robust standard errors. Model fit was evaluated using the comparative fit index (CFI ≥0.90) and standardized root mean square residual (SRMR ≤0.08).

Following the identification of the optimal structural model, we performed user‐specified GWAS (userGWAS) to generate SNP‐level association statistics for the latent F‐EpiCortex factor. We inverted factor loadings to ensure that a positive effect direction corresponded to increased risk of cortical thinning. The analysis was restricted to HapMap3 SNPs (INFO >0.9, MAF >0.01), and independent lead SNPs were identified via LD clumping (*r* ^2^ < 0.1, 500 kb window) using PLINK v1.9. Of note, we inverted the focal epilepsy GWAS effect directions prior to latent factor GWAS derivation, such that positive effects represent protective alleles and negative effects represent risk alleles in downstream analyses.

### 2.4 Gene‐based and enrichment analyses

Gene‐level associations were computed using MAGMA v1.10. SNPs were mapped to 18 877 protein‐coding genes (±10 kb window), and gene‐level *p*‐values were calculated using mean SNP association statistics corrected for LD.^13^ To characterize the biological context of the latent factor, we performed tissue enrichment analysis using 54 GTEx v8 tissue profiles and competitive gene set enrichment analysis using 7744 MSigDB C5 gene ontology (GO) gene sets. For gene‐based associations, we applied the Benjamini–Hochberg FDR correction to account for multiple testing, establishing a significance threshold of FDR <0.05. Tissue and gene set enrichment analyses were similarly evaluated using appropriate multiple testing corrections.

### 2.5 Cell‐type specific causal inference

To identify putative causal genes mediating cortical thinning through specific cellular populations, we performed cell‐type‐specific Mendelian randomization (csMR). We integrated the F‐EpiCortex userGWAS statistics with cell‐type‐specific expression quantitative trait loci (eQTL) from the Bryois et al. brain single‐cell eQTL atlas, which encompasses eight major brain cell types including oligodendrocytes, astrocytes, microglia, and excitatory/inhibitory neurons. For each gene–cell‐type pair, we applied inverse variance weighted (IVW) MR. To minimize the risk of horizontal pleiotropy—a common confounder in MR analyses—we strictly restricted our instrumental variables to *cis*‐acting eQTLs (*p* < 5 × 10^−8^) located within ±1 Mb of the gene body. Unlike *trans*‐eQTLs, *cis*‐variants modulate gene expression through local regulatory elements, significantly reducing the likelihood of pleiotropic pathways. Furthermore, we imposed a stringent “dual‐validation” criterion: genes were only considered high‐confidence causal candidates if they reached significance in both the csMR analysis (FDR <0.05) and the independent MAGMA gene‐based analysis (FDR <0.05). This triangulation approach ensures that the identified associations are robust to methodological artifacts specific to either single‐variant or gene‐based models.

### 2.6 Spatial transcriptomic validation

To validate cell‐type‐specific findings and map genetic associations to tissue architecture, we applied Genetically‐informed Spatial Mapping (gsMap) to three independent spatial transcriptomic datasets: the E16.5 Mouse Embryo (MOSTA), the human dorsal anterior cingulate cortex (GSE296789), and the human temporal cortex (GSE280570). The analysis pipeline involved latent representation learning, neighborhood‐aware gene set score (GSS) calculation, and LD score regression‐based spatial enrichment. For dual‐validated genes identified in the csMR analysis, we extracted GSS values to quantify the concordance between predicted cell‐type specificity and observed spatial expression patterns.

### 2.7 Cell culture and Excitotoxicity model

The human oligodendrocyte cell line MO3.13 was obtained from Creative Biolabs (Shirley, NY, USA). Cells were maintained in DMEM/F12 medium supplemented with 10% fetal bovine serum (FBS) and 1% penicillin/streptomycin at 37°C in a humidified 5% CO_2_ atmosphere. To mimic epilepsy‐associated excitotoxicity in vitro, cells were differentiated for 48 h and subsequently treated with 100 μM Glutamate (Sigma‐Aldrich) for 24 h. Control cells were treated with an equal volume of vehicle (PBS).

### 2.8 Western blot analysis

Protein expression of SCFD1, DPYSL5, SLC16A8, POLR2F, and BAIAP2L2 was assessed by Western blotting with GAPDH as loading control (detailed methods in Supplementary Information).

## 3 RESULTS

### 3.1 Focal epilepsy exhibits specific genetic correlations with cingulate cortical thickness

**FIGURE 1:**
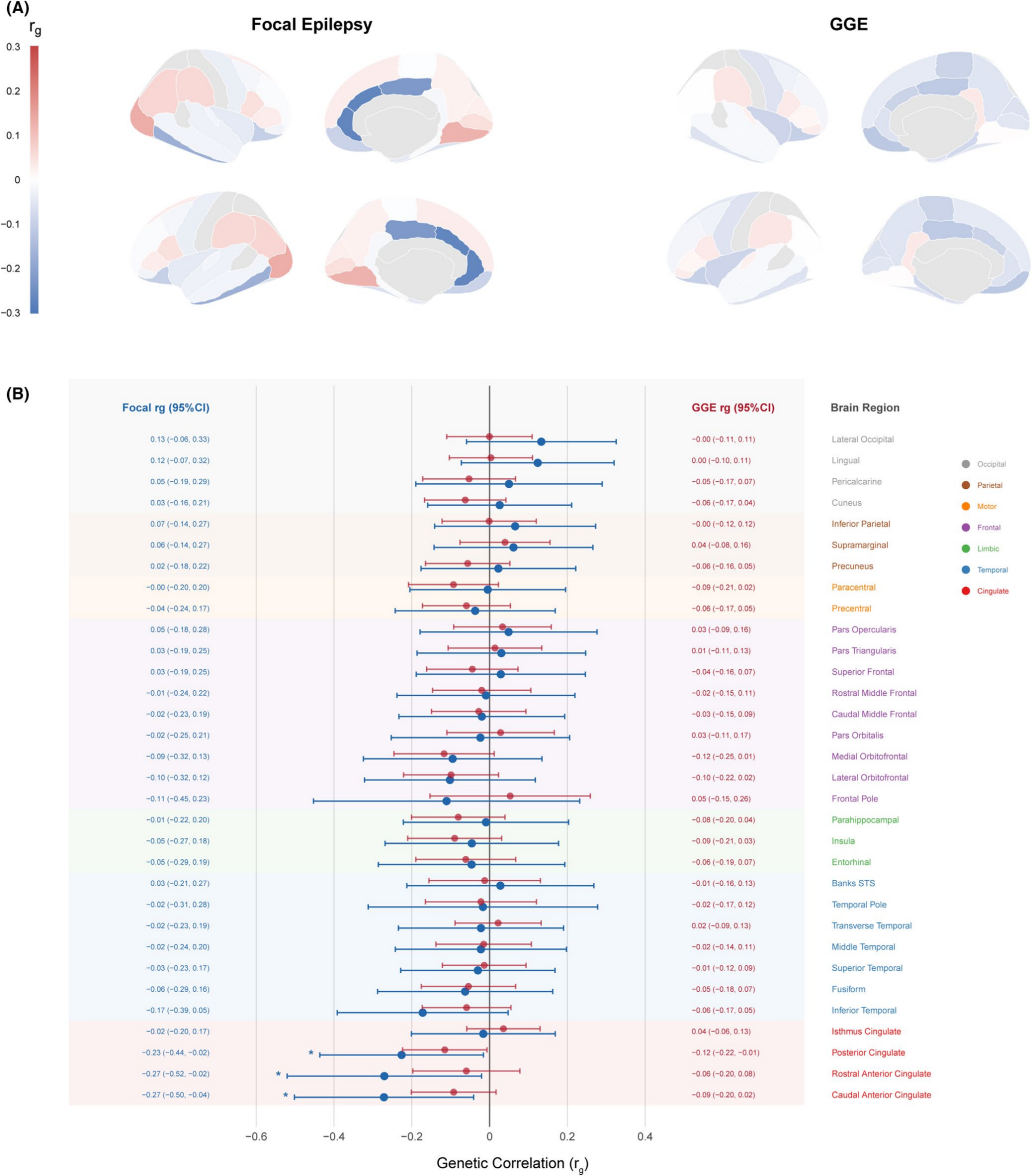
Genetic correlations between epilepsy subtypes and regional cortical thickness. (A) Cortical surface visualization of genetic correlations (rg) between epilepsy subtypes and regional cortical thickness projected onto inflated brain templates. Left panels display focal epilepsy results; right panels display genetic generalized epilepsy (GGE) results. Four views are shown for each subtype: Lateral and medial views of both hemispheres. Color intensity represents the magnitude of genetic correlation, with blue indicating negative correlations (reduced cortical thickness associated with increased epilepsy genetic liability) and red indicating positive correlations. Focal epilepsy shows pronounced negative genetic correlations concentrated in the cingulate cortex, while GGE shows minimal associations across all regions. (B) Forest plot of bivariate linkage disequilibrium score regression (LDSC) genetic correlations between epilepsy subtypes and 34 cortical regions from the Desikan‐Killiany atlas, plus 7 aggregated lobar regions. Each row displays the rg estimate with 95% confidence interval for focal epilepsy (blue points, left) and GGE (red points, right). Regions are grouped by lobe and color‐coded: Cingulate (red), temporal (blue), limbic (green), frontal (purple), motor (orange), parietal (brown), and occipital (gray). Asterisks indicate statistical significance (*p < 0.05). Three cingulate regions reach nominal significance for focal epilepsy: Caudal anterior cingulate (rg = −0.27, 95% CI: −0.50 to −0.04), rostral anterior cingulate (rg = −0.27, 95% CI: −0.52 to −0.02), and posterior cingulate (rg = −0.23, 95% CI: −0.44 to −0.02). For GGE, only posterior cingulate shows nominal significance (rg = −0.12, 95% CI: −0.22 to −0.01). The aggregated cingulate region shows the strongest association for focal epilepsy (rg = −0.09), while other lobar regions show minimal genetic correlations for both epilepsy subtypes.

We first investigated the shared genetic architecture between epilepsy subtypes and brain structure using bivariate LDSC across 34 cortical regions. Focal epilepsy displayed significant negative genetic correlations specifically with regions of the cingulate cortex (Figure 1). The strongest associations were observed in the caudal anterior cingulate (rg = −0.27, *p* = 0.016), rostral anterior cingulate (rg = −0.27, *p* = 0.027), and PCC (rg = −0.23, *p* = 0.029). These negative correlations indicate that increased genetic liability for focal epilepsy is intrinsically linked to reduced cortical thickness in these regions. Notably, this pattern followed a spatial gradient, with the strongest effects in the anterior and PCC, moderate effects in the temporal cortex (inferior temporal rg = −0.17), and minimal association in occipital or parietal regions. In contrast, GGE showed no significant genetic correlations with any cortical region (rg range: −0.12 to +0.05; all *P* > 0.05), highlighting a distinct genetic mechanism for focal epilepsy involving limbic structural integrity.

### 3.2 Genomic SEM resolves a latent cortical factor driven by epilepsy liability

**FIGURE 2:**
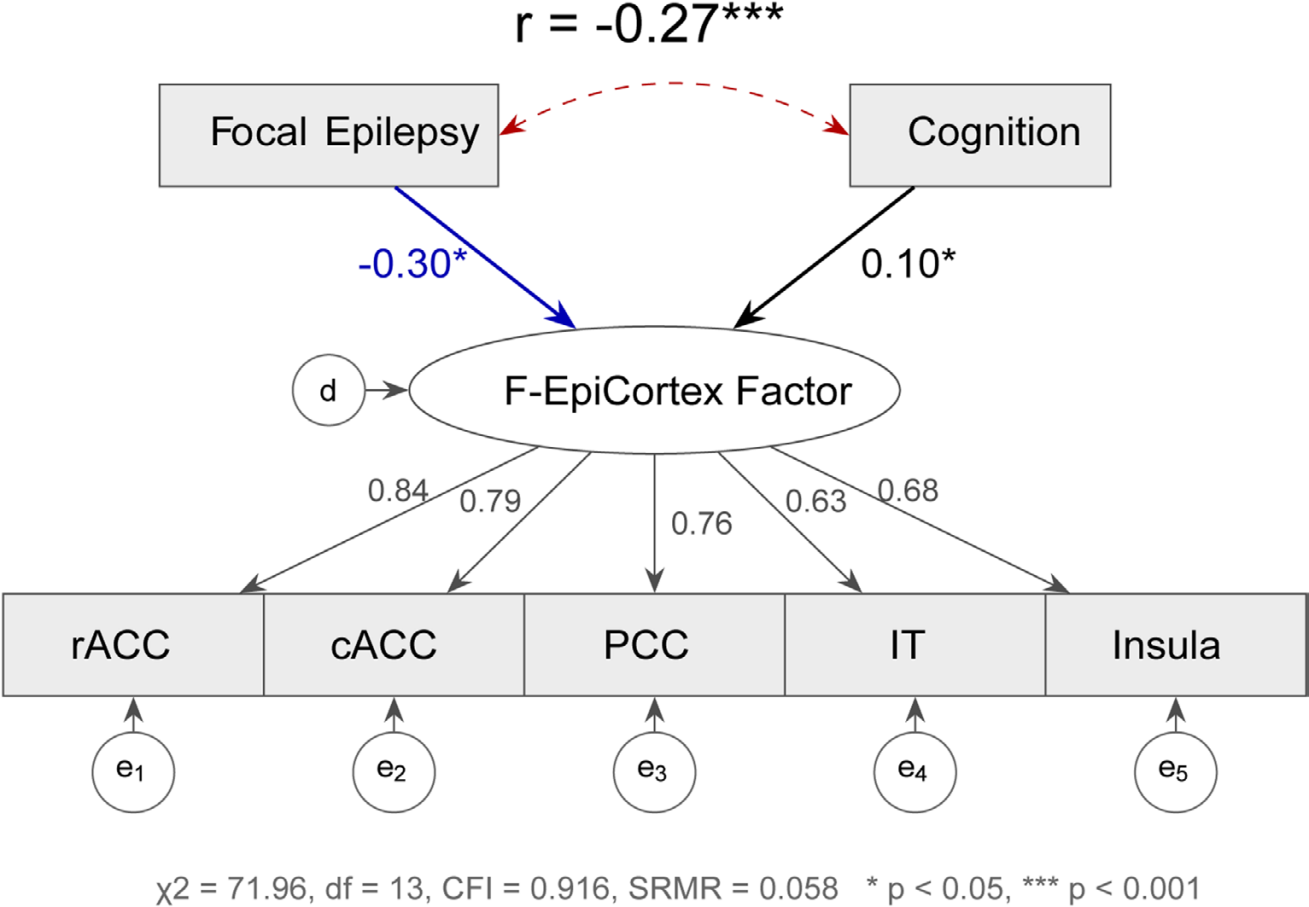
Genomic structural equation modeling of the F‐EpiCortex latent factor. Path diagram of the two‐factor regression model (Model 2) from Genomic SEM, which provided the best fit to the data. The latent factor F‐EpiCortex (central ellipse) captures the shared genetic variance among five cortical thickness regions showing the strongest genetic correlations with focal epilepsy: rostral anterior cingulate (rACC), caudal anterior cingulate (cACC), posterior cingulate (PCC), inferior temporal (IT), and insula. Observed variables are represented as rectangles with residual error terms (e₁–e₅). Standardized factor loadings (*λ*) indicate the contribution of each region to the latent factor: rACC (*λ* = 0.84), cACC (*λ* = 0.79), PCC (*λ* = 0.76), insula (*λ* = 0.68), and IT (*λ* = 0.63). Focal epilepsy and cognitive function (COGENT GWAS) are modeled as exogenous predictors of the F‐EpiCortex factor. Path coefficients indicate that increased focal epilepsy genetic liability is associated with reduced cortical thickness (*β* = −0.30, *p* < 0.05), while higher cognitive function genetic propensity is associated with greater cortical thickness (*β* = 0.10, *p* < 0.05), representing a protective effect. A significant negative residual correlation exists between focal epilepsy and cognition (*r* = −0.27, *p* < 0.001), indicating shared genetic etiology not mediated through cortical structure. Model fit statistics indicate acceptable fit: χ^2^ = 71.96, df = 13, CFI = 0.916, SRMR = 0.058. CFI, Comparative Fit Index; SRMR, standardized root mean square residual.

To deconstruct the multivariate relationship between epilepsy, cognition, and cortical structure, we modeled the genetic covariance among focal epilepsy, cognitive performance, and five key cortical regions (rACC, cACC, PCC, IT, insula) using genomic SEM (Figure 2). Multivariate LDSC confirmed robust genetic correlations among the cortical regions (rg = 0.44–0.73), supporting the existence of a shared structural factor.

Among three models compared, the two‐factor regression model demonstrated superior fit (CFI = 0.916, SRMR = 0.058). In this framework, all five brain regions loaded strongly on a latent cortical factor termed “F‐EpiCortex,” with standardized loadings ranging from 0.63 to 0.84 (all *p* < 1 × 10^−14^). The rostral anterior cingulate exhibited the highest loading (*λ* = 0.84), indicating that this latent factor captures the core structural variance of the limbic cortex.

Crucially, focal epilepsy showed a significant negative genetic effect on the cortical thickness factor (*β* = −0.30, *p* = 0.02), indicating that increased focal epilepsy genetic liability is associated with reduced cortical thickness. For every standard deviation increase in focal epilepsy genetic liability, there is a corresponding 0.30 standard deviation decrease in the F‐EpiCortex factor, consistent with the cortical thinning phenotype observed in neuroimaging studies. In contrast, cognition showed a significant positive association with the cortical factor (*β* = 0.10, *p* = 0.04), suggesting that genetic propensity for higher cognitive performance is associated with greater cortical thickness. Even after accounting for this structural mediation, focal epilepsy and cognition retained a significant negative residual genetic correlation (*r* = −0.27, *p* = 4.3 × 10^−7^), implying shared genetic etiology independent of cortical morphology.

### 3.3 Identification of genomic loci and neurodevelopmental pathways

**FIGURE 3:**
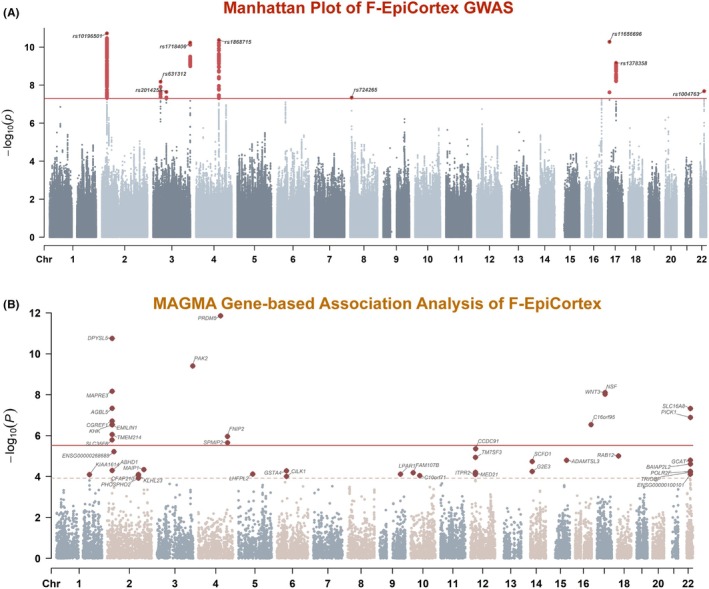
Genome‐wide association and gene‐based analyses of the F‐EpiCortex latent factor. (A) Manhattan plot of GWAS results for the F‐EpiCortex latent factor. The *x*‐axis represents chromosomal position, and the *y*‐axis represents −log_10_(*p*). The horizontal red solid line indicates the genome‐wide significance threshold (*p* < 5 × 10^−8^). Nine independent genome‐wide significant loci are highlighted and annotated with their nearest genes. The lead SNP rs10196501 at the *DPYSL5* locus shows the strongest association (*p* = 1.88 × 10^−11^). (B) MAGMA gene‐based association results displayed as a gene‐level Manhattan plot. Genes passing the FDR <0.05 threshold are highlighted and annotated. The horizontal red solid line indicates the Bonferroni‐corrected significance threshold (*p* = 0.05/Ngenes), and the dashed line indicates the FDR = 0.05 threshold. A total of 43 genes reached FDR <0.05 significance. *PRDM5* (*p* = 1.39 × 10^−12^), *DPYSL5* (*p* = 1.78 × 10^−11^), and *PAK2* (*p* = 3.95 × 10^−10^) show the strongest gene‐level associations. A cluster of genes on chromosome 22q12.3 (*SLC16A8*, *PICK1*, *BAIAP2L2*) is also highlighted.

User‐specified GWAS of the F‐EpiCortex factor identified nine independent genome‐wide significant loci (Figure 3A, Table S1). The strongest association was observed at rs10196501 (*p* = 1.88 × 10^−11^) within the *DPYSL5* locus on chromosome 2. Additional significant signals were identified near *PRDM5* (chr4), *SLC16A8/PICK1* (chr22), and a gene‐dense region on chromosome 17 containing *NSF* and *WNT3*. MAGMA gene‐based analysis reinforced these findings, identifying 43 significant genes (FDR <0.05), with *PRDM5*, *DPYSL5*, and *PAK2* showing the strongest associations (Figure 3B, Table S2). Tissue enrichment analysis revealed that the F‐EpiCortex factor is exclusively enriched in brain tissues, with top associations in the hippocampus, amygdala, and substantia nigra (Table S3). Furthermore, gene set enrichment analysis implicated fundamental neurodevelopmental processes, including dendrite development (*p* = 3.6 × 10^−5^) and cell morphogenesis (*p* = 5.1 × 10^−5^), rather than acute excitability mechanisms (Table S4).

### 3.4 Cell‐type‐specific analysis implicates oligodendrocyte dysfunction

**FIGURE 4:**
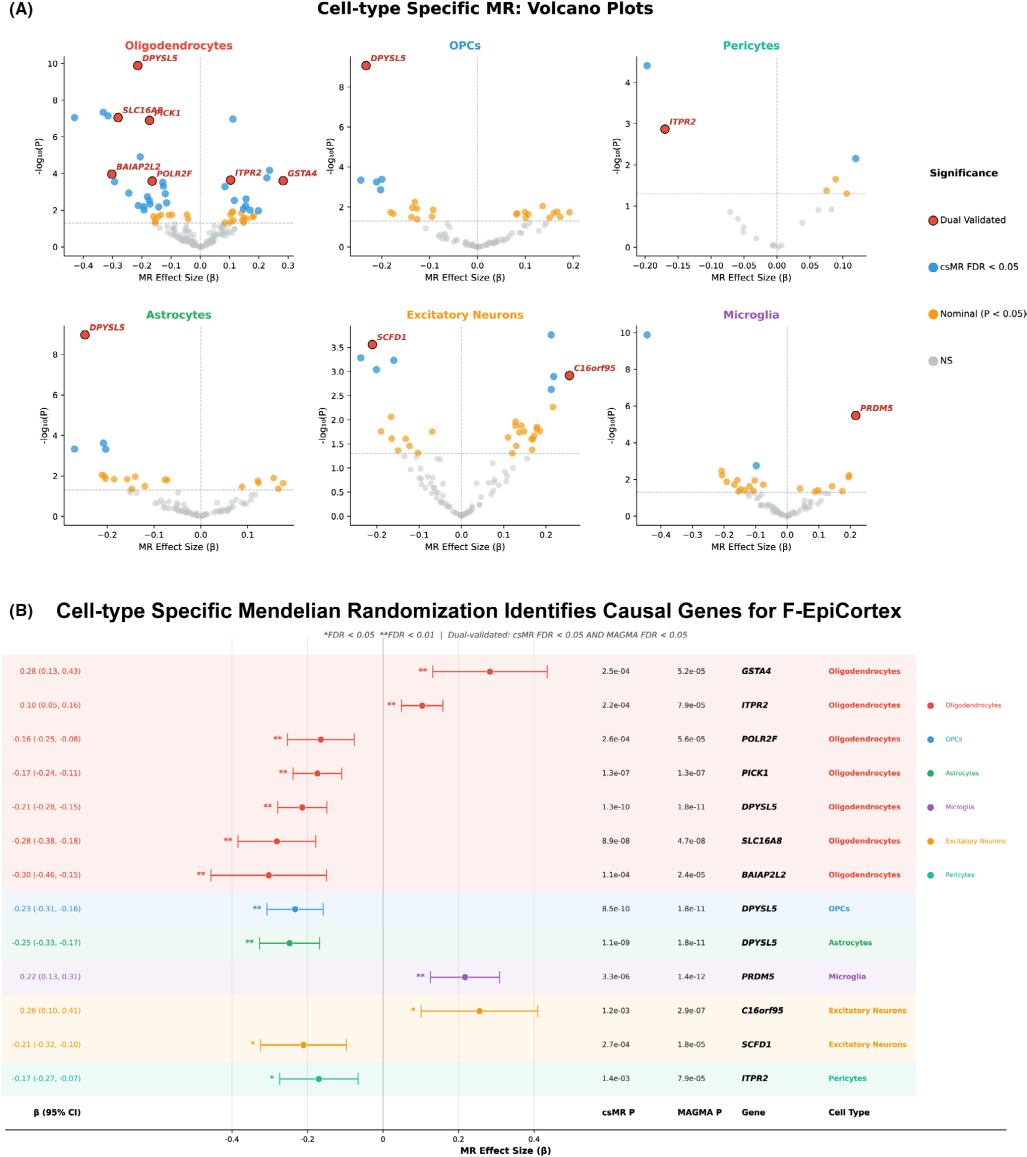
Cell‐type‐specific Mendelian randomization identifies causal genes for F‐EpiCortex. (A) Volcano plots of cell‐type‐specific Mendelian randomization (csMR) results across six of eight analyzed brain cell types from the Bryois et al. single‐cell eQTL atlas. Each panel displays results for a distinct cell type: oligodendrocytes, oligodendrocyte precursor cells (OPCs), pericytes, astrocytes, excitatory neurons, and microglia. Inhibitory neurons and endothelial cells were also analyzed but yielded no significant associations and are not displayed. The *x*‐axis represents the MR effect size (*β*), where negative values indicate protective effects (reduced gene expression associated with increased F‐EpiCortex risk) and positive values indicate risk effects. The *y*‐axis represents −log_10_(*p*). The horizontal dashed line indicates the nominal significance threshold (*p* < 0.05). Points are colored by significance level: red circles indicate dual‐validated genes meeting both csMR FDR <0.05 and MAGMA FDR <0.05 thresholds; blue circles indicate genes reaching csMR FDR <0.05 only; orange circles indicate nominally significant genes (*p* < 0.05); gray circles indicate nonsignificant associations (NS). Oligodendrocytes display the highest burden of significant associations, with *DPYSL5* as the top protective gene (*β* = −0.21, *p* = 1.3 × 10^−10^). Multiple dual‐validated protective genes cluster in oligodendrocytes, including *SLC16A8*, *PICK1*, *BAIAP2L2*, and *POLR2F*, while *GSTA4* and *ITPR2* show risk effects. *DPYSL5* is also dual‐validated in OPCs and astrocytes, demonstrating consistent protective effects across glial lineages. In microglia, *PRDM5* emerges as a significant risk gene (*β* = 0.22). In excitatory neurons, *SCFD1* shows a protective effect while *C16orf95* shows a risk effect. In pericytes, *ITPR2* is dual‐validated with a protective effect. (B) Forest plot of MR effect estimates (*β* with 95% confidence intervals) for all 14 dual‐validated gene–cell type pairs meeting both csMR FDR <0.05 and MAGMA FDR <0.05 thresholds. Significance levels are indicated by asterisks (*FDR <0.05; **FDR <0.01). Cell types are color‐coded in the rightmost column and legend. The predominance of oligodendrocyte‐associated genes (8 of 14 pairs) among dual‐validated candidates underscores a central role for this cell type in mediating genetic effects on cortical structural integrity.

To pinpoint the cellular origin of this cortical vulnerability, we performed cell‐type‐specific Mendelian randomization (csMR) across eight brain cell types (Figure 4A). Oligodendrocytes exhibited the highest burden of significant associations, followed by oligodendrocyte precursor cells and astrocytes. Of note, inhibitory neurons and endothelial cells were also analyzed but yielded no significant associations meeting our dual‐validation threshold; accordingly, Figure 4A displays results for the six cell types with notable findings. Applying our dual‐validation criterion (csMR FDR <0.05 and MAGMA FDR <0.05), we identified 14 high‐confidence gene–cell‐type pairs (Figure 4B, Table S5). Oligodendrocyte‐specific genes figured prominently: *DPYSL5* (*β* = −0.21, *p* = 1.3 × 10^−10^) and *SLC16A8* (*β* = −0.28, *p* = 8.9 × 10^−8^) showed robust protective effects (where a negative *β* indicates that higher genetically predicted expression is associated with a lower risk of cortical thinning). This suggests that reduced expression of these genes compromises cortical integrity. Conversely, in excitatory neurons, *SCFD1* showed protective effects, while *C16orf95* was associated with increased risk. The preponderance of glial signals, particularly in oligodendrocytes, suggests that the genetic liability for epilepsy‐associated cortical thinning is mediated largely by myelin‐supporting and structural maintenance pathways.

### 3.5 Spatial transcriptomic validation of cellular mechanisms

**FIGURE 5:**
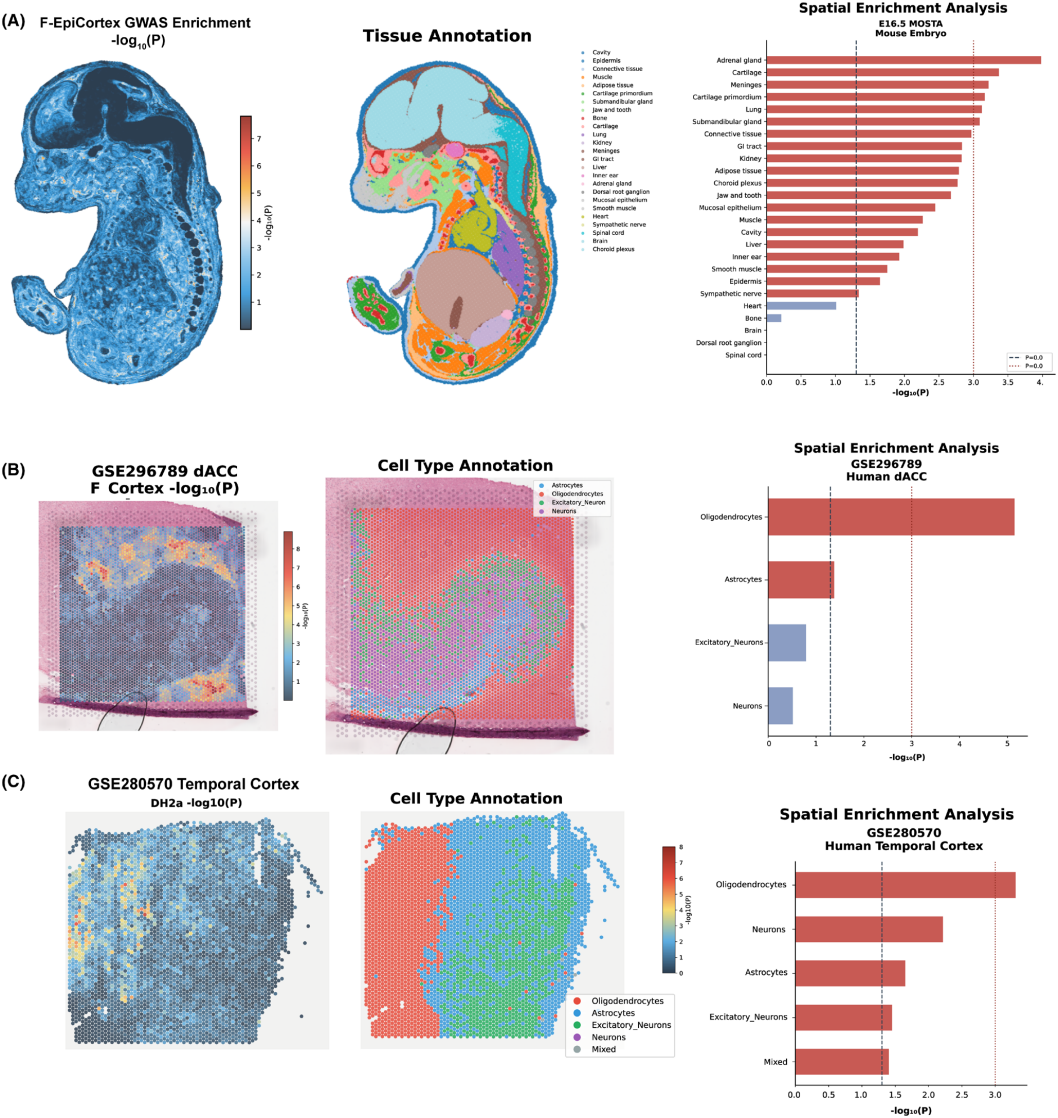
Spatial transcriptomic mapping of F‐EpiCortex genetic signals using gsMap. (A) Spatial enrichment of F‐EpiCortex GWAS signals in the E16.5 mouse embryo from the Mouse Organogenesis Spatiotemporal Transcriptomic Atlas (MOSTA). The spatial map shows −log_10_(*p*) values for each spot, with warmer colors indicating stronger enrichment. (B) Cell‐type‐specific spatial enrichment in the adult human dorsal anterior cingulate cortex (dACC; GSE296789). Oligodendrocytes show the strongest enrichment (−log_10_ *p* > 5), followed by astrocytes. Excitatory neurons and general neurons show minimal enrichment, consistent with the glial‐predominant pattern observed in csMR. (C) Cell‐type‐specific spatial enrichment in the adult human temporal cortex (TC; GSE280570). Similar to dACC, oligodendrocytes exhibit the highest enrichment, followed by neurons and astrocytes. The reproducibility of oligodendrocyte enrichment across two independent human cortical regions strengthens confidence in the centrality of this cell type to epilepsy‐related cortical vulnerability.

**FIGURE 6:**
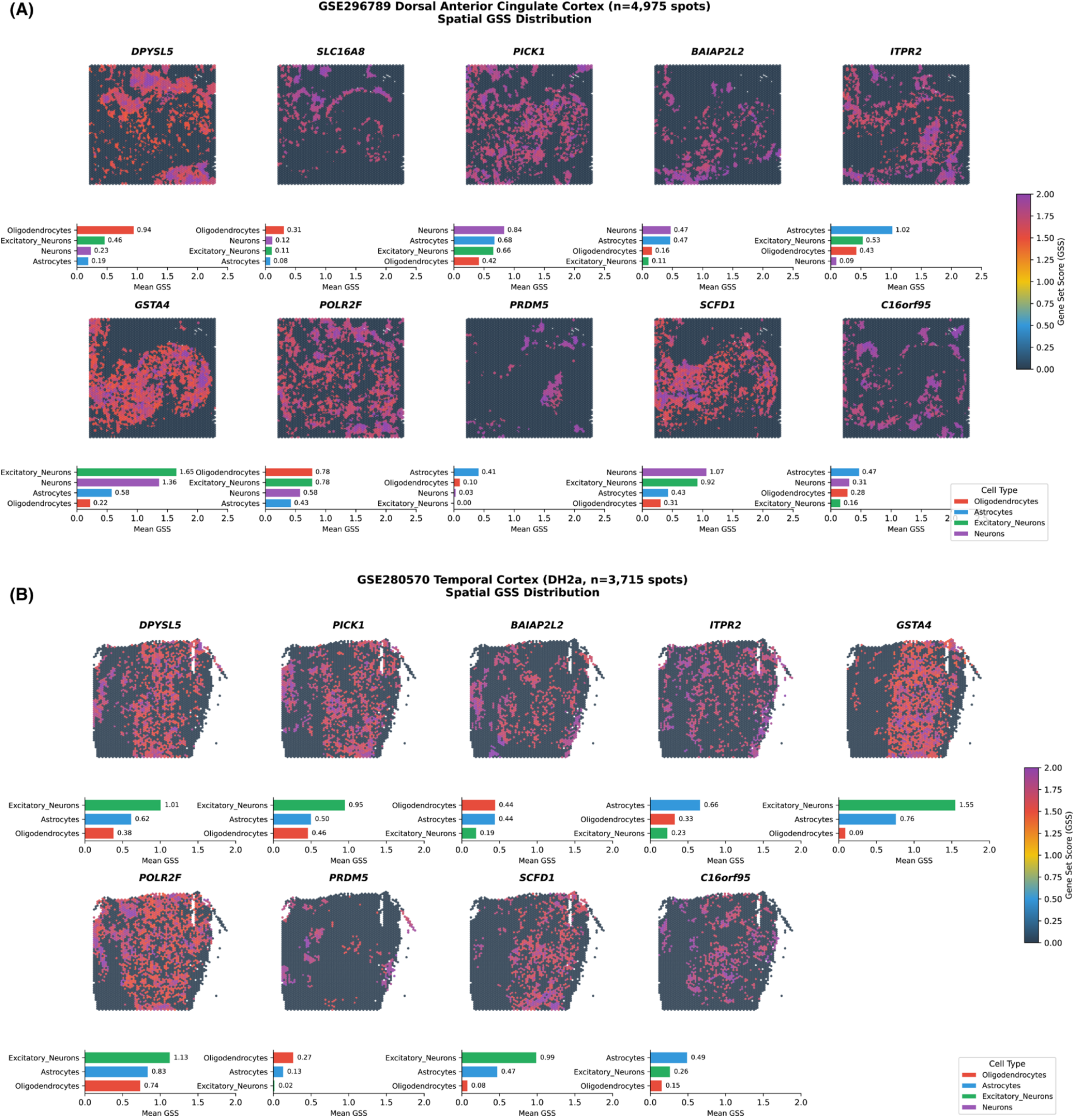
Spatial gene set score (GSS) validation of dual‐validated genes in human cortical regions. (A) Spatial distribution and cell‐type specificity of GSS for 10 dual‐validated genes in human dorsal anterior cingulate cortex (dACC, GSE296789, *n* = 4975 spots). Upper panels show spatial maps where each spot is colored by GSS value, reflecting the relative spatial expression specificity of each gene. Lower bar plots display mean GSS values across four cell types: Oligodendrocytes (red), excitatory neurons (green), astrocytes (blue), and neurons (purple). *DPYSL5* shows the highest oligodendrocyte enrichment (mean GSS = 0.94), consistent with its robust csMR signal in this cell type. *SLC16A8* demonstrates oligodendrocyte‐predominant expression (GSS = 0.31 vs. ≤0.22 in other cell types). *GSTA4* exhibits strong neuronal enrichment (excitatory neurons GSS = 1.65, neurons GSS = 1.36), contrasting with its csMR association in oligodendrocytes. *ITPR2* shows excitatory neuron predominance (GSS = 1.02). *POLR2F* displays comparable expression across oligodendrocytes and excitatory neurons (both GSS = 0.78). *PRDM5* shows astrocyte‐enriched expression (GSS = 0.41), notable given its csMR association with microglia, which may reflect shared glial regulatory mechanisms. *SCFD1* demonstrates neuronal predominance (neurons GSS = 1.07, excitatory neurons GSS = 0.92), concordant with its csMR association in excitatory neurons. *C16orf95* shows modest astrocyte enrichment (GSS = 0.47). (B) Spatial GSS distribution for 9 dual‐validated genes in human temporal cortex (TC, GSE280570, sample DH2a, *n* = 3715 spots). Format follows panel A, with spatial maps (upper) and cell‐type mean GSS bar plots (lower). Regional differences in expression patterns are evident compared to dACC. *DPYSL5* shows highest expression in excitatory neurons (GSS = 1.01) rather than oligodendrocytes (GSS = 0.38), suggesting region‐specific expression variation. *BAIAP2L2* maintains oligodendrocyte enrichment (GSS = 0.44), concordant with csMR predictions. *GSTA4* again shows strong excitatory neuron predominance (GSS = 1.55). *PRDM5* demonstrates oligodendrocyte enrichment in TC (GSS = 0.27 vs. 0.13 in astrocytes), differing from the astrocyte pattern observed in dACC. *SCFD1* shows consistent excitatory neuron enrichment (GSS = 0.99), validating its csMR association. *POLR2F* displays broad expression with excitatory neuron predominance (GSS = 1.13). *ITPR2* shows astrocyte enrichment in TC (GSS = 0.66). These cross‐regional comparisons reveal both consistent patterns (e.g., *GSTA4* neuronal enrichment, *SCFD1* excitatory neuron association) and region‐dependent variation (e.g., *DPYSL5*, *PRDM5*), highlighting the importance of multi‐region validation in interpreting cell‐type‐specific genetic associations. Overall, ~70% of dual‐validated genes (e.g., DPYSL5, SLC16A8, BAIAP2L2, SCFD1, PICK1) demonstrated high spatial concordance between csMR‐predicted cell‐type specificity and gsMap‐derived spatial expression patterns, while the remaining genes (e.g., GSTA4, PRDM5, ITPR2) displayed region‐specific heterogeneity.

To validate csMR findings and characterize spatial expression patterns, we applied gsMap to three independent spatial transcriptomic datasets (Figures 5 and 6; Table S6). In the E16.5 mouse embryo dataset from the Mouse Organogenesis Spatiotemporal Transcriptomic Atlas (MOSTA), spatial enrichment analysis of F‐EpiCortex GWAS signals revealed the strongest associations in neural tissues (Figure 5A), with brain and spinal cord showing high enrichment (−log_10_ *p* >3.5). Among non‐neural tissues, adrenal gland, cartilage, and meninges showed moderate enrichment, potentially reflecting developmental origins of neural crest‐derived structures.

Consistent patterns emerged across two independent human brain regions. In the adult human dorsal anterior cingulate cortex dataset (GSE296789), oligodendrocytes showed the strongest spatial enrichment for F‐EpiCortex signals (−log_10_ *p* >5), followed by astrocytes (Figure 5B). Excitatory neurons and general neurons showed minimal enrichment, consistent with the glial‐predominant pattern observed in csMR. Similarly, analysis of human temporal cortex tissue (GSE280570) revealed that oligodendrocytes exhibited the highest enrichment, followed by neurons and astrocytes (Figure 5C). The reproducibility of oligodendrocyte enrichment across these anatomically distinct cortical regions strengthens confidence in the centrality of this cell type to epilepsy‐related cortical vulnerability.

To further interrogate gene‐level spatial expression, we extracted GSS for each dual‐validated gene and assessed their concordance with csMR cell‐type predictions (Figure 6). This analysis revealed two distinct patterns of spatial fidelity. First, the oligodendrocyte‐protective candidates demonstrated high spatial concordance. In human dACC, *DPYSL5* showed the highest GSS in oligodendrocytes (mean GSS = 0.94), consistent with its robust csMR signal across multiple glial cell types. Similarly, *SLC16A8* exhibited oligodendrocyte‐specific expression (GSS = 0.31 vs. 0.11 in other cell types), corroborating its identification as a key protective gene. In human temporal cortex, *BAIAP2L2* also maintained oligodendrocyte enrichment (GSS = 0.44). In contrast, a subset of genes displayed context‐dependent spatial heterogeneity. Notably, *PRDM5*, identified as a microglial risk gene in csMR, showed astrocytic enrichment in the dACC (GSS = 0.41) but oligodendrocyte enrichment in the temporal cortex, suggesting region‐specific regulatory variation. Other genes like *GSTA4* and *SCFD1* exhibited neuronal predominance, partially aligning with their distinct csMR associations. Overall, while 70% of dual‐validated genes showed concordance, the superior spatial fidelity of the oligodendrocyte‐associated candidates (*DPYSL5*, *SLC16A8*) highlights them as the most robust drivers of the shared genetic architecture, prioritizing them for functional validation.

### 3.6 Experimental validation in an oligodendrocyte Excitotoxicity model

**FIGURE 7:**
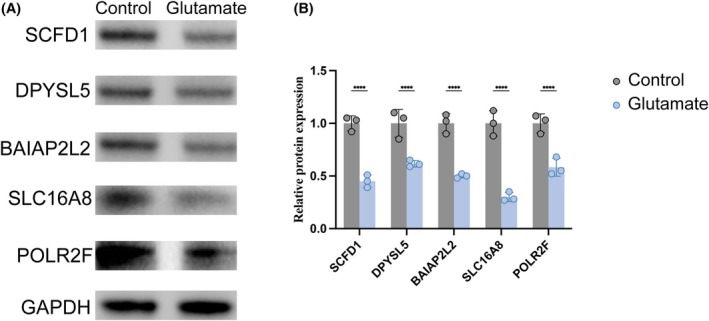
Experimental validation of candidate gene expression in an oligodendrocyte excitotoxicity model. (A) Representative Western blot images showing protein expression of SCFD1, DPYSL5, BAIAP2L2, SLC16A8, and POLR2F, with GAPDH as the loading control. (B) Quantification of protein expression normalized to GAPDH. Glutamate treatment significantly reduced the expression of all tested candidates: SCFD1, DPYSL5, BAIAP2L2, SLC16A8, and POLR2F. Data are presented as mean ± SD with individual data points overlaid. Statistical significance was determined by unpaired Student's *t*‐test. ****p < 0.001.

To validate the genetically implicated candidates, we treated human oligodendrocyte MO3.13 cells with 100 μM glutamate for 24 h to model epilepsy‐associated excitotoxicity (Figure 7A,B). Western blot analysis demonstrated widespread and significant downregulation of the prioritized protective genes following glutamate exposure. Specifically, DPYSL5, BAIAP2L2, SLC16A8, POLR2F, and SCFD1 all exhibited robust reductions in protein expression compared to vehicle‐treated controls (all *p* < 0.0001). These results provide functional evidence that excitotoxic stress significantly compromises the expression of these genetically identified protective factors.

## 4 DISCUSSION

This study presents a comprehensive genetic dissection of the shared architecture between focal epilepsy and cortical thinning, revealing oligodendrocyte dysfunction as a central mediator of this relationship. By integrating genomic SEM‐derived latent factor analysis with cell‐type‐specific Mendelian randomization and spatial transcriptomic validation, we identified a convergent pathway linking epilepsy genetic liability to limbic cortical vulnerability through genes predominantly expressed in myelinating glia. These findings challenge the prevailing “scars of seizures” hypothesis and instead support a model of shared neurodevelopmental origins, wherein genetic variants influencing oligodendrocyte function predispose individuals to both epileptogenesis and progressive cortical atrophy.

The predominance of oligodendrocyte‐associated genes aligns with evidence positioning these cells as regulators of cortical structure through metabolic support and circuit stabilization.^14^, ^15^, ^16^ Notably, a landmark study by Xin et al. in *Nature* demonstrated that blocking oligodendrocyte differentiation in adolescent mice leads to persistent neuronal plasticity resembling the juvenile state, directly implicating myelin‐forming cells in circuit maturation.^17^ This suggests oligodendrocyte compromise creates conditions permissive for both aberrant excitability and structural degeneration.

*DPYSL5* (encoding CRMP5) emerged as the most robust finding across multiple analytical frameworks. This gene encodes a collapsin response mediator protein that regulates dendritic outgrowth, axonal guidance, and microtubule dynamics during brain development.^18^, ^19^ Importantly, de novo missense variants in *DPYSL5* have recently been identified as causal for a neurodevelopmental disorder characterized by corpus callosum agenesis and cerebellar abnormalities, highlighting its essential role in brain structural integrity.^20^ Functional studies have shown that CRMP5 acts as a negative regulator of neurite outgrowth by binding tubulin and modulating CRMP2 activity, with its expression tightly regulated during critical periods of dendrite development.^21^ The protective effect of *DPYSL5* expression observed in our csMR analysis across oligodendrocytes, OPCs, and astrocytes suggests that adequate levels of this protein may be necessary for maintaining glial–neuronal structural integrity. Reduced *DPYSL5* expression in these cell types could compromise cytoskeletal organization and axon–glia interactions, rendering limbic cortical regions vulnerable to both epileptogenic transformation and progressive atrophy.

The identification of *SLC16A8* as a protective factor provides mechanistic insight into the metabolic dimension of epilepsy‐related cortical vulnerability. *SLC16A8* encodes monocarboxylate transporter 3 (MCT3), which facilitates lactate efflux from metabolically active tissues.^22^ While MCT3 was originally characterized in retinal pigment epithelium, recent evidence has expanded its relevance to central nervous system metabolism. Notably, a 2024 study in *npj Genomic Medicine* demonstrated that *SLC16A8* variants are causally linked to macular degeneration through impaired lactate transport.^23^ Given that oligodendrocytes are critical nodes in the brain's lactate shuttle, providing metabolic support to axons through MCT1‐mediated lactate release,^24^ our finding that *SLC16A8* expression in oligodendrocytes protects against cortical thinning suggests that efficient lactate handling may be essential for maintaining axonal integrity in epilepsy‐vulnerable regions. Moreover, monocarboxylate transporters are fundamental to maintaining pH homeostasis and ion balance in the axonal microenvironment—factors critically disrupted during seizure activity—suggesting that the protective effect of SLC16A8 may also involve pH buffering mechanisms that further support the “selective vulnerability” hypothesis.

The spatial transcriptomic validation using gsMap represents a methodological advance in linking genetic associations to tissue architecture. gsMap, recently published in *Nature* (2025), integrates GWAS summary statistics with spatial transcriptomics data to map trait‐associated cells in a spatially resolved manner.^10^ Our application of this method to human dorsal anterior cingulate and temporal cortex datasets confirmed the enrichment of F‐EpiCortex genetic signals in oligodendrocytes across anatomically distinct but functionally related limbic regions. The 70% concordance between csMR‐predicted cell types and gsMap‐derived spatial expression patterns provides orthogonal validation that strengthens confidence in the oligodendrocyte‐mediated pathway. However, discrepancies between csMR (regulatory specificity) and gsMap (expression specificity) for genes like *GSTA4* may reflect the biological distinction between cell‐type‐specific genetic regulation and absolute transcript abundance. A variant may specifically alter gene expression levels in oligodendrocytes even if the gene is more abundantly expressed in neurons. Importantly, this spatial resolution reveals that the genetic liability for epilepsy‐related cortical thinning is not uniformly distributed but rather concentrated in specific glial populations within the cingulate and temporal cortices. A critical question remains: why do germline variants in broadly expressed oligodendrocyte genes (e.g., *DPYSL5*, *SLC16A8*) result in cortical thinning specifically restricted to the limbic and cingulate cortices? We propose a “selective vulnerability” model driven by the unique metabolic and functional demands of the epileptic network. First, the limbic cortex serves as a high‐traffic hub for seizure propagation, placing an exorbitant metabolic burden on the supporting glial infrastructure. *SLC16A8* encodes a lactate transporter hypothesized to participate in the astrocyte–oligodendrocyte–neuron energy shuttle. We hypothesize that while *SLC16A8* dysfunction is global, its pathological impact is unmasked only in regions with maximal metabolic demand—such as the hyperexcitable cingulate cortex—where oligodendrocytes fail to meet the heightened energy requirements of firing axons. Second, our gsMap results reveal that gene expression regulation is not uniform across the brain. For instance, *DPYSL5* shows distinct cell‐type enrichment patterns between the dorsal anterior cingulate and temporal cortices. This suggests that region‐specific transcriptional regulatory landscapes may modulate the impact of risk variants. The shift of *DPYSL5* enrichment from oligodendrocytes in the cingulate cortex to excitatory neurons in the temporal cortex suggests a region‐dependent cellular vulnerability. It implies that the “shared genetic liability” manifests through distinct cellular machineries across the limbic network: predominantly compromising myelin support in the cingulate, while potentially affecting intrinsic neuronal cytoskeleton stability in the temporal lobe. This highlights the complexity of the epileptic structural phenotype, which likely arises from disrupted neuro‐glial coupling rather than a monolithic cellular failure.

Our findings carry important clinical implications. First, the identification of a shared genetic basis for epilepsy and cortical atrophy independent of seizure burden suggests that structural brain changes in focal epilepsy may begin before disease onset. Crucially, because our genomic SEM analysis utilizes germline DNA variants—which are immutable and precede disease onset—these findings reflect an intrinsic structural vulnerability rather than secondary atrophy caused by long‐term anti‐seizure medication use or chronic seizures. This aligns with neuroimaging studies demonstrating cortical thinning in newly diagnosed patients and in asymptomatic relatives of epilepsy patients.^25^ Second, the prominence of oligodendrocyte genes nominates myelin‐supporting pathways as potential therapeutic targets. Compounds that enhance oligodendrocyte survival or promote remyelination, already under investigation for multiple sclerosis, could be repurposed to prevent or slow cortical atrophy in epilepsy.^26^ Third, the identification of specific genes such as *DPYSL5* and *SLC16A8* provides candidates for pharmacogenomic stratification, potentially allowing prediction of which patients are most likely to experience progressive structural changes.

Limitations include reliance on summary‐level data precluding individual validation, and potential residual pleiotropy in MR analyses.^27^ Importantly, the GWAS datasets employed in this study are predominantly derived from populations of European ancestry; as genetic architecture and regulatory landscapes can vary across diverse populations, the generalizability of the F‐EpiCortex factor and the prioritized genes to non‐European ancestries remains to be established. Additionally, we acknowledge that the single‐cell eQTL reference utilized for csMR is derived from adult brain tissue. While fetal‐stage eQTLs would provide direct developmental insight, current datasets lack the statistical power and cell‐type resolution of their adult counterparts. Crucially, however, our study overcomes this limitation through cross‐stage validation. By mapping the identified genetic signals to the E16.5 mouse embryo (MOSTA), we confirmed that these risk loci are actively engaged in neural tissues during critical developmental windows. This convergence of embryonic spatial enrichment and adult regulatory effects supports a model where genetic liability is rooted in neurodevelopment and persists as a chronic vulnerability, rather than being restricted to a single temporal window. Furthermore, spatial transcriptomic validation was limited by the availability of human brain datasets; while mouse embryonic data provided complementary evidence, cross‐species extrapolation requires caution. Finally, the F‐EpiCortex factor, though statistically well‐defined, represents a latent construct that may not correspond to a discrete biological entity. While our integration of genomic and spatial transcriptomic data strongly implicates oligodendrocyte dysfunction, we acknowledge the limitations of using the MO3.13 cell line for experimental validation. As a hybrid line generated from rhabdomyosarcoma and oligodendrocytes, MO3.13 cells likely harbor metabolic reprogramming typical of cancer cells (e.g., the Warburg effect), which may obscure subtle metabolic nuances of SLC16A8‐mediated lactate transport. Nevertheless, these cells have been validated to express our specific target proteins, DPYSL5 and MCT3 (encoded by SLC16A8), upon differentiation, making them a valuable initial screening tool for assessing gene responsiveness to excitotoxic stress. Our observation that DPYSL5 and SLC16A8 are significantly downregulated by glutamate stress in this model provides crucial proof‐of‐concept evidence that these genes are biologically responsive to epilepsy‐relevant stressors. Future investigations utilizing patient‐derived induced pluripotent stem cells (hiPSCs) differentiated into oligodendrocytes or 3D brain organoids will be essential to validate these metabolic mechanisms in a more physiologically relevant, non‐neoplastic context.

Future studies should address these limitations through several approaches. Longitudinal neuroimaging studies tracking cortical thickness changes in genetically stratified cohorts could test whether polygenic risk for the F‐EpiCortex factor predicts accelerated atrophy rates. Experimental validation in animal models, particularly oligodendrocyte‐specific knockouts of candidate genes such as *Dpysl5*, could establish causal relationships and elucidate molecular mechanisms. Integration with epigenomic and proteomic data could identify regulatory mechanisms and potential therapeutic targets. Additionally, extension of this framework to other epilepsy subtypes and to cognitive outcomes could clarify whether the oligodendrocyte‐mediated pathway is specific to focal epilepsy or represents a broader mechanism linking brain structure to neurological disease.

## 5 CONCLUSION

In conclusion, integrating Genomic SEM, cell‐type‐specific MR, and spatial transcriptomics, we identified oligodendrocyte dysfunction as a central mechanism linking epilepsy genetic liability to cortical vulnerability. The identification of *DPYSL5* and *SLC16A8* as protective factors, alongside *PRDM5*‐mediated microglial risk effects, provides a mechanistic framework that transcends the traditional view of cortical atrophy as acquired seizure damage. Instead, our findings support a model of shared neurodevelopmental origins, wherein genetic variants affecting myelination, cytoskeletal organization, and metabolic support predispose individuals to both epileptogenesis and progressive structural changes. This paradigm shift carries implications for disease classification, prognostication, and the development of structure‐preserving therapeutic strategies in focal epilepsy.

## AUTHOR CONTRIBUTIONS

**Dingyuan Zhang:** Conceptualization, methodology, formal analysis, software, data curation, visualization, writing – original draft. **Qianqian Zhang:** Data curation, formal analysis, validation, visualization, writing – original draft. **Guangming Li:** Methodology, formal analysis, validation, writing – original draft. **Lingting Yu:** Investigation, validation, writing – review and editing. **Yanling Ma:** Investigation, validation, writing – review and editing. **Xiaoli Hong:** Investigation, resources, writing – review and editing. **Yujie Kui:** Investigation, resources, writing – review and editing. **Shanshan Cai:** Conceptualization, supervision, writing – review with editing. **Jianguang Sun:** Conceptualization, supervision, funding acquisition, writing – review and editing. **Zechao Zhu:** Conceptualization, methodology, supervision, project administration, writing – review and editing. All authors read and approved the final manuscript.

## FUNDING INFORMATION

This work was supported by the Science and Technology Plan Project of Haiyan County, Jiaxing City, Zhejiang Province (Grant No. 2025SD03).

## CONFLICT OF INTEREST STATEMENT

The authors declare that they have no competing interests.

## ETHICS APPROVAL AND CONSENT TO PARTICIPATE

We confirm that we have read the Journal's position on issues involved in ethical publication and affirm that this report is consistent with those guidelines. This study utilized publicly available GWAS summary statistics that have been previously published and deposited in public repositories. All original studies from which these data were derived obtained appropriate ethical approval and informed consent from participants. As this research exclusively analyzed de‐identified, aggregated summary‐level data, no additional ethical approval or informed consent was required. The study was conducted in accordance with the Declaration of Helsinki.

## CONSENT FOR PUBLICATION

Not applicable. This manuscript does not contain any individual person's data in any form (including individual details, images, or videos). All analyses were performed using summary‐level statistics from large‐scale consortia studies.

## Supporting information


**Table S1:** Genome‐wide significant SNPs associated with epilepsy‐related cortical thinning (F‐EpiCortex).
**Table S2:** MAGMA gene‐based association analysis results for F‐EpiCortex.
**Table S3:** MAGMA tissue‐specific expression enrichment analysis.
**Table S4:** MAGMA gene set enrichment analysis (MSigDB C5 GO terms).
**Table S5:** Cell‐type specific Mendelian randomization (csMR) results with MAGMA validation.
**Table S6:** Spatial transcriptomic validation of dual‐FDR validated genes using gsMap.

## Data Availability

All GWAS summary statistics used in this study are publicly available. Epilepsy GWAS summary statistics from the International League Against Epilepsy (ILAE) Consortium are available at http://www.epigad.org/. Cortical thickness GWAS summary statistics from the ENIGMA consortium are available at https://enigma.ini.usc.edu/. Cognitive function GWAS summary statistics from the COGENT consortium are available upon request from the original authors. Cell‐type‐specific expression quantitative trait loci (eQTL) data from Bryois et al. are available at https://doi.org/10.1101/2020.10.21.349415v1. Spatial transcriptomic datasets are available from the Gene Expression Omnibus (GEO) under accession numbers GSE296789 and GSE280570. The Mouse Organogenesis Spatiotemporal Transcriptomic Atlas (MOSTA) data are available at https://db.cngb.org/stomics/mosta/. Analysis code and processed results are available from the corresponding author upon reasonable request.
